# Optimal Landmark for Chest Compressions during Cardiopulmonary Resuscitation Derived from a Chest Computed Tomography in Arms-Down Position

**DOI:** 10.3390/jcdd9040100

**Published:** 2022-03-27

**Authors:** Pimpan Usawasuraiin, Borwon Wittayachamnankul, Boriboon Chenthanakij, Juntima Euathrongchit, Phichayut Phinyo, Theerapon Tangsuwanaruk

**Affiliations:** 1 Department of Emergency Medicine, Faculty of Medicine, Chiang Mai University, Chiang Mai 50200, Thailand; pimpan.u@cmu.ac.th (P.U.); borwon.witt@cmu.ac.th (B.W.); boriboon.c@cmu.ac.th (B.C.); 2 Department of Radiology, Faculty of Medicine, Chiang Mai University, Chiang Mai 50200, Thailand; juntima.eua@cmu.ac.th; 3 Department of Family Medicine, Faculty of Medicine, Chiang Mai University, Chiang Mai 50200, Thailand; phichayut.phinyo@cmu.ac.th; 4 Center for Clinical Epidemiology and Clinical Statistics, Faculty of Medicine, Chiang Mai University, Chiang Mai 50200, Thailand

**Keywords:** basic cardiac life support, cardiac arrest, cardiopulmonary resuscitation, computed X-ray tomography, emergency department

## Abstract

Compressions at the left ventricle increase rate of return of spontaneous circulation. This study aimed to identify the landmark of the point of maximal left ventricular diameter on the sternum (LVmax) by using chest computed tomography (CCT) in the arms-down position, which was similar to an actual cardiac arrest patient. A retrospective study was conducted between September 2014 and November 2020. We included adult patients who underwent CCT in an arms-down position and measured the rescuer’s hand. We measured the distance from the sternal notch to LVmax (DLVmax), to the lower half of sternum (DLH), and to the point of maximal force of hand, which placed the lowest palmar margin of the rescuer’s reference hand at the xiphisternal junction. Thirty-nine patients were included. The LVmax was located below the lower half of the sternum; DLVmax and DLH were 12.6 and 10.0 cm, respectively (*p* < 0.001). Distance from the sternal notch to the point of maximal force of the left hand, with the ulnar border located at the xiphisternal junction, was close to DLVmax; 11.3 and 12.6 cm, respectively (*p* = 0.076). In conclusion, LVmax was located below the lower half of the sternum, which is recommended by current guidelines.

## 1. Introduction

Out-of-hospital cardiac arrest is a common occurrence [1]. Only 5.1% of patients receive chest compressions according to basic life support (BLS) and 8.3% have a return of spontaneous circulation (ROSC). Survival until hospital discharge is 4.12% [2]. Therefore, high-quality cardiopulmonary resuscitation (CPR) is very crucial. High-quality CPR, including defibrillation in a patient with shockable rhythms, will provide organ perfusion, improve coronary perfusion pressure during cardiac arrest, and ROSC [3,4,5,6]. Previous studies performed chest compressions in different positions to improve the hemodynamic effect or to compress the left ventricle [7,8,9,10,11]. Chest compressions at the left ventricle increase the coronary perfusion pressure, improve hemodynamics, and rate of ROSC in the swine model [7]. If the aorta is compressed, the blood’s resistance will be increased. As a result, the amount of blood generated by the left ventricle decreases, as does cardiac output [12].

The current 2020 American Heart Association (AHA) for cardiopulmonary resuscitation and emergency cardiovascular care guidelines, 2021 European Resuscitation Council (ERC) Guidelines, and 2020 Korean guidelines recommend performing chest compressions by placing the heel of hand at the lower half of the sternum [13,14,15,16]. Previous studies used chest computed tomography (CCT) or transesophageal echocardiography (TEE). They found that beneath the lower half of the sternum was the ascending aorta and the left ventricular outflow tract while beneath the junction of the lower end of sternum and cartilage (xiphisternal junction or sternoxiphoid junction) was the left ventricle [12,17]. Based on the concept of compressions at the point of maximal left ventricular diameter, several studies were conducted by using a CCT to identify the optimal landmark for chest compressions. In both healthy and cardiac disease patients, the position of the left ventricular on the sternum derived from a CCT was located below the currently recommended lower half of the sternum [8,9,10,18,19,20]. 

In the past, some studies used CCT to identify the position of the left ventricle on the sternum, and they did not mention the position of the patient’s arms. In general, patients who underwent CCT were usually performed in a standard arms-up position (arms above the head) to minimize the artifact on the imaging output. In contrast, cardiac arrest patients usually underwent CPR in an arms-down position (arms by the side of the torso) due to loss of muscular tone, or the rescuer repositioned patients’ arms for convenience for chest compression or transfer. Different arm positions and respiratory phases affected the location of the left ventricle [9,17,21]. According to the present data, the study of the optimal position for chest compressions derived from CCT has significant limitations in terms of arms position and respiratory phases. The use of the CCT in an arms-down position will be similar to an actual cardiac arrest patient.

This study aimed to identify the optimal landmark of the maximal left ventricular diameter on the sternum (LVmax) for chest compressions and to propose a new hand placement using CCT in the arms-down position.

## 2. Materials and Methods

### 2.1. Study Design

We conducted a retrospective, cross-sectional study at Maharaj Nakorn Chiang Mai Hospital, a university-based, tertiary care hospital, between September 2014 and November 2020. 

The study was conducted according to the guidelines of the Declaration of Helsinki, and the protocol was approved by the Research Ethics Committee of the Faculty of Medicine, Chiang Mai University (No. 219/2020) on 15 June 2020. For the rescuer’s hand measurement, informed consent was obtained from all participants before they participated in the study. For the patient’s CCT measurement, written informed consent was waived due to the retrospective design. This study was prospectively registered in the Thai Clinical Trials Registry (TCTR20200825002). We followed the Strengthening the Reporting of Observational Studies in Epidemiology (STROBE) statement.

### 2.2. Selection of Participants

For the patient’s CCT measurement, we included the CCT in the arms-down position of non-cardiac arrest adult patients (age ≥ 18 years old) who were scanned for several purposes. Individuals with cardiothoracic anomalies such as destroyed lung, lobectomy, atelectasis, hiatal hernia, mediastinal or lung mass, >5 mm-depth pericardial effusion, and pericardial tumor or cyst were excluded from the study [10]. Destroyed lung is the destruction of the lobe or total lung presented as cavities, fibrosis or bronchial dilatation, or stenosis [22,23,24].

For the rescuer’s hand measurement, we recruited BLS providers who had an opportunity to perform CPR. Adults (age ≥ 18 years old) without hand abnormalities such as amputation, fracture, or acromegaly were included to obtain hand data.

### 2.3. Methods of Measurement

#### 2.3.1. Rescuer’s Hand Measurement

Both participants’ hands were measured for the width of the hand’s heel which distributed maximal force at two centimeters above the distal end of the carpal bone. Based on a previous study, we used the point of maximal force while performing chest compressions that was 68.6% and 73% from the radial border of right and left hands, respectively [25]. The width of the radial border’s heel to the point of maximal force was then measured with a standard tape measure (Figure S1 in Supplementary Materials). From the participant’s dominant hand, we measured the width of one, two, and three fingerbreadths at the distal interphalangeal joint of the index finger to adjust with the hand placement. For precision, there were repeated measurements three times, and the average was represented for each parameter. Hand measurement data were analyzed alongside the cardiothoracic parameters from CCT measurement.

#### 2.3.2. Patient’s CCT Measurement

All included patients received CCT in an arm-down position using multidetector-row CT Platforms with standard technique (SOMATOM Definition, and SOMATOM Force, Siemens Healthcare GmbH, Forchheim, Germany) with three-dimensional (3D) reconstruction (off-line workstation Syngo, Siemens Healthcare GmbH, Erlangen, Germany). The cardiothoracic parameters in CCT were retrieved and reviewed with the Picture Archiving and Communication System (Synapse Radiology PACS, version 5.7.000, FUJIFILM Medical System, Lexington, MA, USA). All images were measured and interpreted by an experienced board-certified thoracic radiologist.

**Figure 1:**
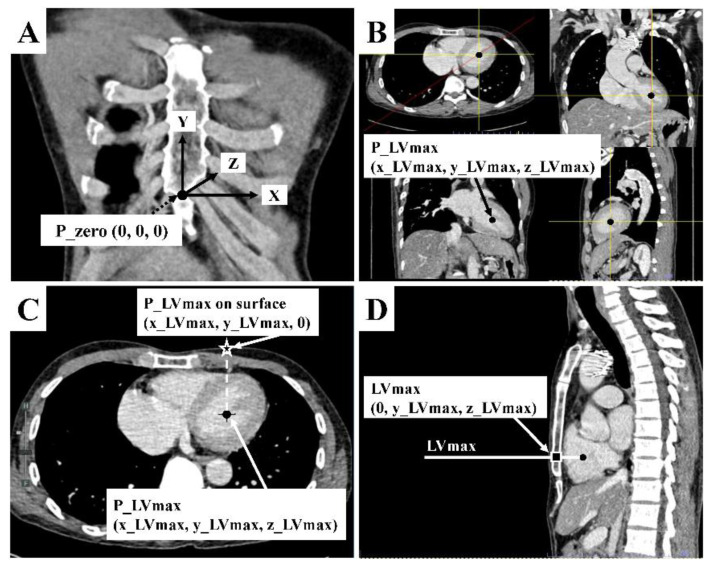
Chest computed tomography measurement. (**A**) The zero point (P_zero: [0, 0, 0]) was defined as the midpoint of the xiphisternal junction. It is the palpable lower end of the sternum where all sternal body, xiphoid process, and costal margins meet. From P_zero, the leftward, upward, and into the thorax directions of the patient were designated as positive on x, y, and z axes, respectively, which formed right angles to one another. (**B**) The P_LVmax (x_LVmax, y_LVmax, z_LVmax) was the midpoint of maximal left ventricular diameter in coronal, sagittal, and axial view. Its 3D coordinate was identified by using the picture archiving and communicating system’s function. (**C**) Assuming that P_LVmax was located on the anterior chest surface (Z = 0) just vertically above that midpoint, defined as P_LVmax on the surface (x_LVmax, y_LVmax, 0). (**D**) The sagittal view of CCT was used to locate the maximal left ventricular diameter on the sternum, defined as LVmax (0, y_LVmax, z_LVmax).

We modified the 3D coordinate system of Cho S. et al. [18] on CCT to identify the point of maximal left ventricular diameter on the sternum (LVmax) (Figure 1) according to the CPR guidelines that suggest performing chest compressions over the sternum to minimize adverse injuries [15].

After we obtained the means of the rescuer’s hand measurement earlier, P_Force was a distance from the rescuer’s hand heel to the point of maximal force of hand on the compression surface. The rescuer’s left hand was measured from the heel’s radial border to the point of maximal force of hand (P_Force_LR) and from the heel’s ulnar border to the point of maximal force hand (P_Force_LU) while the left hand was compressing on the surface. Similarly, the rescuer’s right hand was measured from the heel’s radial border to the point of maximal force of hand (P_Force_RR) and from the heel’s ulnar border to the point of maximal force of hand (P_Force_RU) while the right hand was compressing on the surface. 

**Figure 2:**
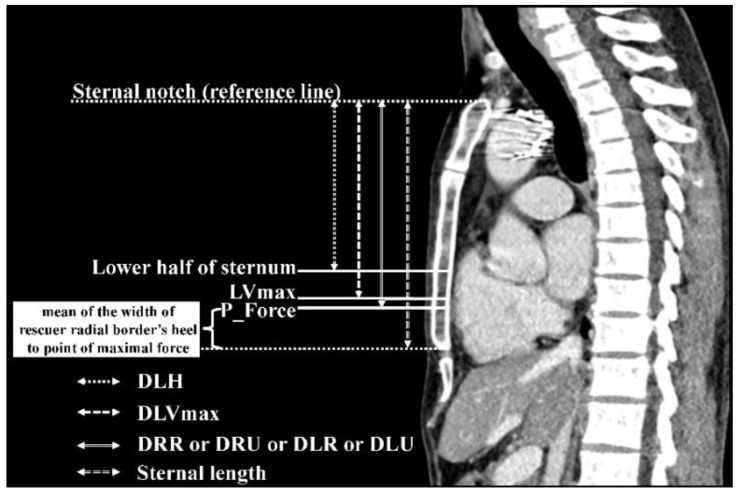
Sagittal view of chest computed tomography. P_Force indicated the point of the maximal chest compression force of the rescuer’s hand heel which placed the lowest palmar margin of the rescuer’s hand at the xiphisternal junction (lower end of the sternum). Abbreviations: DLH, distance from the sternal notch to the lower half of sternum; DLVmax, distance from the sternal notch to the point of maximal left ventricular diameter on the sternum; DLR, distance from the sternal notch to the point of maximal force of the rescuer’s left hand with the radial border located at the xiphisternal junction; DLU, distance from the sternal notch to the point of maximal force of the rescuer’s left hand with the ulnar border located at the xiphisternal junction; DRR, distance from the sternal notch to the point of maximal force of the rescuer’s right hand with the radial border located at the xiphisternal junction; DRU, distance from the sternal notch to the point of maximal force of the rescuer’s right hand with the ulnar border located at the xiphisternal junction; LVmax, The point of maximal left ventricular diameter on the sternum.

Figure 2 showed that using the P_Force of the rescuer’s hand of four hand-placement scenarios (P_Force_LR, P_Force_LU, P_Force_RR, P_Force_RU) incorporated with the patient’s sternal notch on CCT (as a reference point) to comprise the four types of hand placement as the following. (1) DLR was a distance from the sternal notch to the point of maximal force of the rescuer’s left hand with the radial border located at the xiphisternal junction when the rescuer approached the patient’s right side and put the rescuer’s left hand down on the patient’s chest. (2) DLU was the distance from the sternal notch to the point of maximal force of the rescuer’s left hand with the ulnar border located at the xiphisternal junction when the rescuer approached the patient’s left side and put the rescuer’s left hand down on the patient’s chest. (3) DRR was the distance from the sternal notch to the point of maximal force of the rescuer’s right hand with the radial border located at the xiphisternal junction when the rescuer approached the patient’s left side and put the rescuer’s right hand down on the patient’s chest. (4) DRU was the distance from the sternal notch to the point of maximal force of the rescuer’s right hand with the ulnar border located at the xiphisternal junction when the rescuer approached the patient’s right side and put the rescuer’s right hand down on the patient’s chest. Therefore, P_Force became the point of the maximal chest compression force of the hand, which placed the lowest palmar margin (radial or ulnar border depended on those types) of the rescuer’s reference hand at the patient’s xiphisternal junction (lower end of the sternum).

As the sternal notch was defined as a reference point in the CCT, we measured the distance from the sternal notch to the lower half of the sternum (DLH). DLVmax was a distance from the sternal notch to the point of maximal left ventricular diameter on the sternum. Moreover, to find the optimal landmark for chest compressions, we adjusted the hand placement by placing the lowest palmar margin of the reference hand one, two, or three fingerbreadths below the xiphisternal junction.

### 2.4. Data Collection

The baseline characteristics including sex, age, height, weight, body mass index (BMI), comorbidity, respiration, and cardiothoracic parameters were obtained from patients who underwent CCT. The rescuer’s hand measurements were obtained of the rescuer’s hand and fingerbreadth width, the width of the hand’s heel, and the width of radial border’ heel to point of maximal force.

### 2.5. Outcome Measures

The primary outcome was to identify the optimal landmark for chest compressions on the sternum which would directly compress the LVmax.

### 2.6. Sample Size Estimation

The sample size was based on statistical power on the primary outcome. A pilot study was conducted on six patients to measure CCT for cardiothoracic parameters. The DLVmax was 12.7 ± 1.7 cm. Our expert consensus accepted the differences of DLVmax and real chest compressions’ point on sternum less than 15% to conclude as the near point. Estimated sample size for comparison of means was used which mean 1 = 13 cm, mean 2 = 11 cm (15% difference of mean 1), standard deviation = 1.7 cm, α error of 0.05 (two-sided), power of 80% and 15% dropout rate. The required sample size for the CCT measurement was 39 patients. 

For the rescuer’s hand parameter, a previous study showed the width of the hand’s heel was 8.3 ± 0.6 cm and 7.3 ± 0.4 cm in right and left, respectively [25]. Estimation of population mean was used which standard deviation of right hand = 0.6 cm, left hand = 0.4 cm, precision of 0.2, α error of 0.05 (two-sided), power of 80% and 15% dropout rate. We estimated that the required sample size for the rescuer’s right hand and left hand was 45 and 23. Therefore, we required the rescuer’s hand measurement to be 60.

### 2.7. Data Analysis

Baseline characteristics were presented as number, percentage, mean, standard deviation, median, and interquartile range (IQR) as appropriate. Data visualization was used to determine the normal distribution of continuous variables. Categorical data were assessed using Fisher’s exact test. DLVmax, DLH, and distance from the sternal notch to P_force (DRR, DRU, DLR, or DLU) as continuous data were compared by paired t-test or Wilcoxon signed-rank test as appropriate. A two-sided significance level of *p* < 0.05, and 95% confidence intervals (CI) were considered statistically significant. No statistical significance was noted between DLVmax and DRR, DRU, DLR, or DLU, the point was acceptably closed to each other. LVmax and P_force were also considered with this criterion. We also presented a modified Bland–Altman plot for the difference between DLVmax and DRR, DRU, DLR, or DLU. The missing data were handled with multiple imputation methods. The Stata version 16 (Stata Corp LLC, College Station, TX, USA) was used for statistical analysis. 

## 3. Results

### 3.1. Characteristics of Participants

Thirty-nine patients were included to measure the cardiothoracic parameters in CCT (Table 1) with twenty-two males (56.4%) and a median age of 67 years (IQR 49–78 years). The median BMI was 23.3 kg/m^2^ (IQR 19.7–25 kg/m^2^). Among them, 30 (76.9%) were inspiratory hold. 

A total of 69 rescuer participants were included in the hand measurements (Table 1). There were 37 males (53.6%) with a median age of 26 years (IQR 24–31 years). The median BMI was 22.3 kg/m^2^ (IQR 20–26.2 kg/m^2^). Most of the rescuer participants were lay rescuers with a BLS certification (26.1%), registered nurses (23.2%), paramedics (15.9%), and medical students (15.9%). Among them, 62 (90%) were right-handed. In particular, the mean of one fingerbreadth was 1.9 ± 0.3 cm and two fingerbreadths were 3.6 ± 0.4 cm.

In the rescuer’s hand measurement, the width of the hand’s heel was not different in right and left hand (mean ± standard deviation: 7.9 ± 0.9 cm and 7.9 ± 0.8 cm, *p* = 0.431). However, the width of the radial border’ heel to point of maximal force was significantly different in each hand (mean ± standard deviation: 5.4 ± 0.6 cm and 5.8 ± 0.6 cm, *p* < 0.001) (Table S1 in Supplementary Materials).

### 3.2. Primary Outcome

The distances from the sternal notch to the different points on the sternum in CCT measurement are shown in Table 2. The DLVmax was neither different in inspiratory hold nor expiratory hold (mean ± standard deviation: 12.1 ± 1.9 cm and 12.9 ± 2.7 cm, *p* = 0.386). Also, the sternal length and DLH were not different (*p* = 0.441 and *p* = 0.334, respectively).

**Figure 3:**
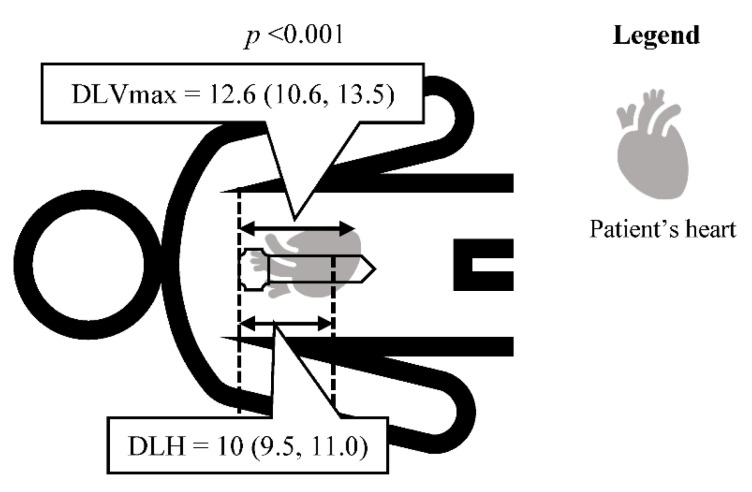
Distance from the sternal notch to the point of maximal left ventricular diameter on the sternum versus lower half of sternum. The distances showed as median (interquartile range) in centimeters. Differences were analyzed with Wilcoxon signed-rank test. Abbreviations: DLVmax, distance from the sternal notch to the point of maximal left ventricular diameter on the sternum; DLH, distance from the sternal notch to the lower half of sternum.

The median of DLVmax and DLH were 12.6 cm (IQR 10.6–13.5) and 10 cm (IQR 9.5–11.0) (*p* < 0.001) (Figure 3). The DLVmax and DLH were significantly different. This result indicated LVmax was located lower than the lower half of the sternum.

**Figure 4:**
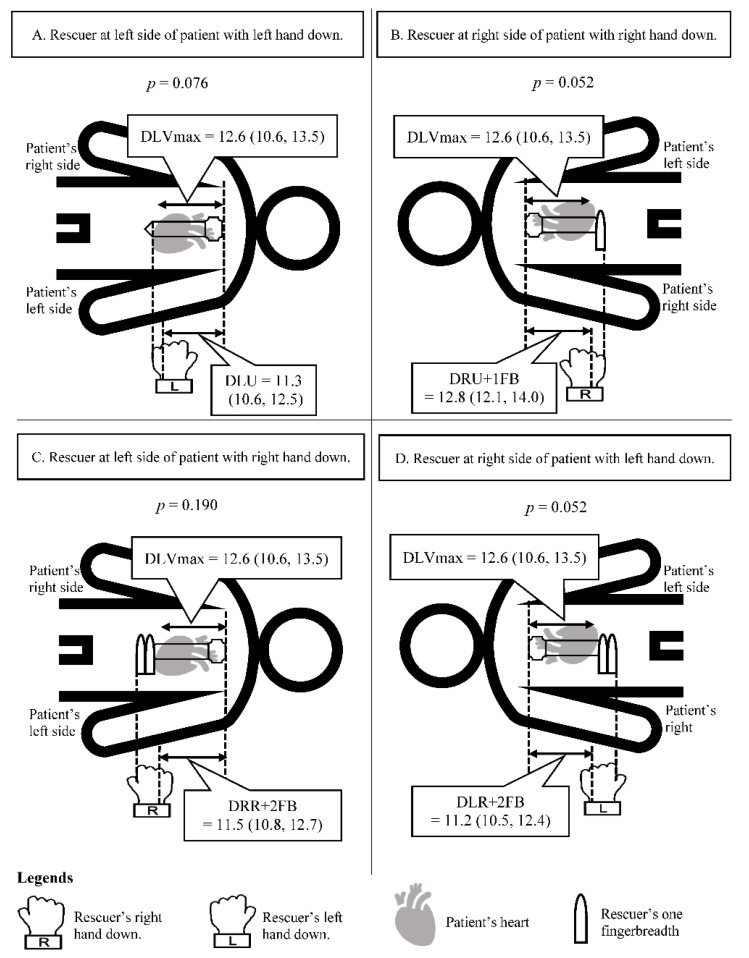
Optimal landmarks for chest compressions. The distances showed as median (interquartile range) in centimeters. Differences were analyzed with Wilcoxon signed-rank test. (**A**) Rescuer at left side of patient with left hand down. (**B**) Rescuer at right side of patient with right hand down. (**C**) Rescuer at left side of patient with right hand down. (**D**) Rescuer at right side of patient with left hand down. Abbreviations: DLVmax, distance from the sternal notch to the point of maximal left ventricular diameter on the sternum; DLU, distance from the sternal notch to the point of maximal force of the rescuer’s left hand with the ulnar border located at the xiphisternal junction; DRU + 1FB, distance from the sternal notch to the point of maximal force of the rescuer’s right hand with the ulnar border located at below the xiphisternal junction one fingerbreadth; DRR + 2FB, distance from the sternal notch to the point of maximal force of the rescuer’s right hand with the radial border located at below the xiphisternal junction two fingerbreadths; DLR + 2FB, distance from the sternal notch to the point of maximal force of the rescuer’s left hand with the radial border located at below the xiphisternal junction two fingerbreadths; 95% CI, 95% confidence interval.

We used cardiothoracic and rescuer’s hand parameters to analyze the difference between DLVmax and DRR, DRU, DLR, or DLU. We found DLU was not significantly different from DLVmax; the median of DLVmax and DLU were 12.6 cm (10.6, 13.5 cm) and 11.3 cm (10.6, 12.5 cm) (*p* = 0.076). Moreover, we adjusted the hand placement by using one and two fingerbreadths to find the optimal landmark for chest compressions. As a result, DLVmax was not significantly different from DRU + 1FB (*p* = 0.052), DRR + 2FB (*p* = 0.190) and DLR + 2FB (*p* = 0.052) (Figure 4). Other hand placement methods with or without fingers adjustment were significantly different from DLVmax and we also presented a modified Bland–Altman plot for differences between DLVmax and DRR, DRU, DLR, or DLU (Figures S2–S5 in Supplementary Material).

## 4. Discussion

The result of this study demonstrated the location of LVmax which is the optimal point for chest compressions. We found LVmax was located lower than the lower half of the sternum, which is recommended by the current CPR guidelines [13,15,16], around 2.5 cm and above the xiphisternal junction around 1.2 cm. 

Previous studies used different positions to improve the hemodynamic effect and found chest compressions at the left ventricle increase the coronary perfusion pressure, improve hemodynamics, and rate of ROSC [7,8,9,10,11]. The 2020 AHA, the 2021 ERC, and the 2020 Korean guidelines recommended performing chest compressions over the sternum to minimize adverse consequences and to place the heel of the hand at the lower half of the sternum [13,14,15,16]. We, therefore, suggest that the optimal point for chest compressions is the LVmax. Moreover, the results of this study showed the LVmax was located lower than the lower half of the sternum which is recommended in the current CPR guidelines. This result is similar to several previous studies derived from a CCT in both healthy and cardiac disease patients [8,9,10,19,20]. However, in obesity (BMI > 30 kg/m^2^), LVmax was located more cephalad than in normal-weight [26]. Cha K.C. et al. also showed that beneath the xiphisternal junction was the location of the left ventricle from CCT [17]. However, the current CPR guidelines recommend the lower half of the sternum as a landmark for chest compression based on a few studies and use the level of carbon dioxide which is released at the end of an exhaled breath (end-tidal carbon dioxide; ETCO_2_) to evaluate the quality of CPR [14,15,27]. In 2013, Cha KC. et al. found that chest compressions at the lower end of the sternum which the center of the heel of the dominant hand placed at the xiphisternal junction resulted in more ETCO_2_ and peak arterial pressure than the nipple line [11]. While, Qvigstad E. et al. found that the different hand placements had no significant difference in ETCO_2_ values during CPR in cardiac arrest patients [27]. According to the present studies, the lower half of the sternum is likely to be recommended as a simple and easy method to perform although the evidence is unclear. These several pieces of evidence support our study that compressions at the LVmax will potentially have a good prognosis in cardiac arrest patients [7,8,9,10,11]. 

This is the study to demonstrate optimal landmark for chest compressions derived from a CCT in an arms-down position which is similar to the position of cardiac position of arrest patient, unlike other studies. The difference in the position of arms may affect the location of the left ventricle. Although we recruited both inspiratory hold and expiratory hold CCT with arm-down position, we found that the DLVmax and DLH were neither different in inspiratory hold nor expiratory hold. According to our study, we imply that the LVmax is optimal for chest compressions in cardiac arrest patients during either assisted or non-assisted ventilation. However, a previous study used the CCT in inspiration with an arms-up position and found that the sternum moved upward [21]. Later, in comparison of the different arms positions during CCT, the result showed the left ventricle in expiration with the arms-down position located in the middle of sternum and was higher than inspiration with the arms-up position [28]. 

Furthermore, our study focused on establishing the simplest and easiest optimal landmark for chest compressions which not only healthcare providers but also bystander CPR could perform in a witnessed arrest situation. The current CPR guidelines have no definite term of hand during chest compression and the present data about whether the dominant or non-dominant hand should be underneath is still unclear [13,14,15,16]. In this study, we found four different landmarks that will directly compress at the LVmax during CPR. For the simplest and easiest method, we propose that the new hand placement for chest compressions is placing the left hand down and the lowest hand’s border (ulnar border) located at the xiphisternal junction (lower end of the sternum) while approaching the left side of the patient. Moreover, we adjusted the hand placement by using one and two fingerbreadths to find the optimal landmark for chest compressions. As a result, first, with a rescuer at the right side of the patient, either placing the right hand down and the lowest hand’s border (ulnar border) located at below the xiphisternal junction one fingerbreadth or placing the left hand down and the lowest hand’s border (radial border) located at below the xiphisternal junction two fingerbreadths. Second, a rescuer at the left side of the patient places the right hand down and the lowest hand’s border (radial border) located below the xiphisternal junction two fingerbreadths (Figure 4).

No studies have reported the effective generation of cardiac output and the critical outcomes such as neurological outcome, survival, or ROSC. The complication of compressions at the LVmax is unclear. Further investigations are needed to address these topics.

## 5. Limitations

This study has some limitations. First, some included CCTs were scanned in unintended expiratory phases which might not be in full expiratory hold. These may be similar to out-of-hospital cardiac arrest patients while performing BLS but may affect the situation of passive full inspiration and expiration from advanced airway. Second, we assumed that the measured parameters for both CCT and hand measurement were in a straight plane line. In a previous study, the rescuer’s hand’s heel was identified by stamping onto a flat paper to determine the area of maximal force while compressing [25]. This area might not always be accurate when compressed on the sternum which is not flat. However, the current CPR guidelines have an unclear term used to describe a hand’s heel and whether the right or left hand should be underneath [13,14,15,16]. Third, the size of a rescuer’s hand varies based on their race. All the participants in our study were Asian. Further study into the width of rescuer’s hands in different races may be a concern for accurate chest compressions. Fourth, the left ventricle could be differently located in the patient with the enlarged left ventricle, which could move leftward away from the midline. However, we are not prespecified to exclude an enlarged left ventricle patient. Moreover, we attempted to include the adult in various conditions at risk of cardiac arrest and used minimal exclusion for generalizability. Fifth, the sample size of our study was slightly small. However, the patients’ CCT and rescuers’ hands met their sample size estimation. CCT was usually obtained in a standard arms-up position. Therefore, our strength was using the CCT in an arms-down position similar to an actual cardiac arrest patient. Prospective design or CCT in an arms-down position primarily for research might be investigated in further study. Sixth, all patients who were measured CCT parameter in our study were Asians. Generally, most Asians are of shorter stature compared to the western population, and this issue might impact the results. Comparing each body’s landmarks among various large populations is still a gap of knowledge.

## 6. Conclusions

The result of this study showed the location of LVmax in the arms-down position to be lower than the guideline-recommended landmark. To optimize chest compression, we suggest placing the left hand down and the lowest hand’s border (ulnar border) located at the lower end of the sternum while staying on the left side of the patient. For the new landmark, outcomes and complications should be evaluated in further investigation.

## Presentation

An earlier version of the abstract was presented as an ePoster entitled “Optimal landmark for chest compressions during cardiopulmonary resuscitation derived from a chest computed tomography in arms-down position” at the conference of ESC Asia 2021 with APSC and AFC (virtual edition) held on 2–4 December 2021. The earlier version of the abstract was published in the Eur. Heart J. 2022; 43 (Supplement_1): ehab849.100. doi:10.1093/eurheartj/ehab849.100.

## Figures and Tables

**Table 1:** Baseline characteristics.

Characteristics	Overall
Patient characteristics (*n* = 39)	
Age—year ^†^	67 (49, 78)
Male—*n* (%)	22 (56.4)
Weight—kg *	61.6 ± 14.8
Height—cm *	161.4 ± 8.5
Body mass index (BMI)—kg/m^2 †^	23.3 (19.7, 25)
Inspiratory hold—*n* (%)	30 (76.9)
Co-morbidity—*n* (%)	
Hypertension	22 (56.4)
Coronary artery disease	10 (25.6)
Dyslipidemia	10 (25.6)
Diabetes	9 (23.1)
End-stage kidney disease	9 (23.1)
Others	24 (61.5)
None	7 (18)
Rescuer’s hand measurement (*n* = 69)	
Age—year ^†^	26 (24, 31)
Male—*n* (%)	37 (53.6)
Weight—kg ^†^	64 (56, 75)
Height—cm *	167.3 ± 7.3
Body mass index (BMI)—kg/m^2 †^	22.3 (20, 26.2)
Career—*n* (%)	
Certified-BLS Lay rescuer	18 (26.1)
Registered nurse	16 (23.2)
Paramedic	11 (15.9)
Medical student	11 (15.9)
Physician	6 (8.7)
Practical nurse	4 (5.8)
Nurse aid	2 (2.9)
Emergency nurse practitioner	1 (1.5)
Right-hand as dominant hand—*n* (%)	62 (90)
Preferred left hand underneath when performed chest compressions—*n* (%)	41 (59.4)
Fingerbreadth width of dominant hand—cm *	
One FB (index finger)	1.9 ± 0.3
Two FB (index, middle fingers)	3.6 ± 0.44
Three FB (index, middle, ring fingers)	5.4 ± 0.6

Abbreviations: BLS, basic life support; FB, fingerbreadth. * mean ± standard deviation. ^†^ median (interquartile range).

**Table 2:** Distances from sternal notch to the different points on sternum.

Measurement (cm)	Overall (*n* = 39)	Inspiratory Hold (*n* = 30)	Expiratory Hold (*n* = 9)	Difference	95% CI	*p*-Value
DLVmax ^†^	12.6 (10.6, 13.5)	12.4 (10.6, 13.4)	12.7 (10.8, 15)	NA	NA	0.527
Sternal length *	13.8 ± 1.6	13.7 ± 1.5	14.3 ± 2	−0.6	−2.2 to 1	0.441
DLH ^†^	10 (9.5, 11)	9.89 (9.5, 10.8)	10.6 (10.2, 11.7)	NA	NA	0.334

Abbreviations: DLVmax, distance from the sternal notch to the point of maximal left ventricular diameter on the sternum; Sternal length, distance from the sternal notch to the xiphisternal junction; DLH, distance from the sternal notch to the lower half of sternum; NA, not applicable; 95% CI, 95% confidence interval. * mean ± standard deviation and analyzed with unpaired *t*-test with unequal variance. ^†^ median (interquartile range) and analyzed with Wilcoxon rank-sum test.

## Data Availability

The datasets are available from the corresponding author on reasonable request. The data are not publicly available due to privacy restrictions.

## Supplementary Materials

The following supporting information can be downloaded at: https://www.mdpi.com/article/10.3390/jcdd9040100/s1, Table S1: Measurement of rescuer’s hand; Figure S1: Rescuer’s hand measurement; Figure S2: Optimal landmarks for chest compressions; Figure S3: Optimal landmark for chest compressions: Add one fingerbreadth below the xiphisternal junction; Figure S4: Optimal landmark for chest compressions: Add two fingerbreadths below the xiphisternal junction; Figure S5: Summary of the optimal landmark for chest compressions by modified Bland-Altman plots.
