# Supplementary material for: Optimal Landmark for Chest Compressions during Cardiopulmonary Resuscitation Derived from a Chest Computed Tomography in Arms-Down Position
Source: J Cardiovasc Dev Dis. 2022 Mar 27;9(4):100. doi: 10.3390/jcdd9040100 (PMC9033090; doi:10.3390/jcdd9040100)
Supplement: Supplementary file 1 [file jcdd-09-00100-s001.zip › jcdd-1624126-supplementary.pdf]

Supplementary Materials

Title: Optimal Landmark for Chest Compressions During Cardiopulmonary Resuscitation Derived from a Chest Computed Tomography in Arms-Down Position

TABLE OF CONTENTS

	Page
**Table S1** Measurement of Rescuer's Hand.	2
**Figure S1** Rescuer's Hand Measurement.	3
**Figure S2** Optimal Landmarks for Chest Compressions.	4
**Figure S3** Optimal Landmark for Chest Compressions: Add one fingerbreadth below the xiphisternal junction.	6
**Figure S4** Optimal Landmark for Chest Compressions: Add two fingerbreadths below the xiphisternal junction.	8
**Figure S5** Summary of the Optimal Landmark for Chest Compressions by Modified Bland-Altman Plots.	10

Table S1 Measurement of Rescuer's Hand

Measurement (centimeter)*	Right hand (n = 69)	Left hand (n = 69)	Difference	95% CI	p-value
Width of hand's heel	7.9 ± 0.9	7.9 ± 0.8	0	-0.1 to 0.1	0.431
Width of radial border' heel to point of maximal force	5.4 ± 0.6	5.8 ± 0.6	0.3	0.3 to 0.4	<0.001

Abbreviations: *95% CI*, 95% confidence interval.

* mean ± standard deviation and analyzed with paired t-test.

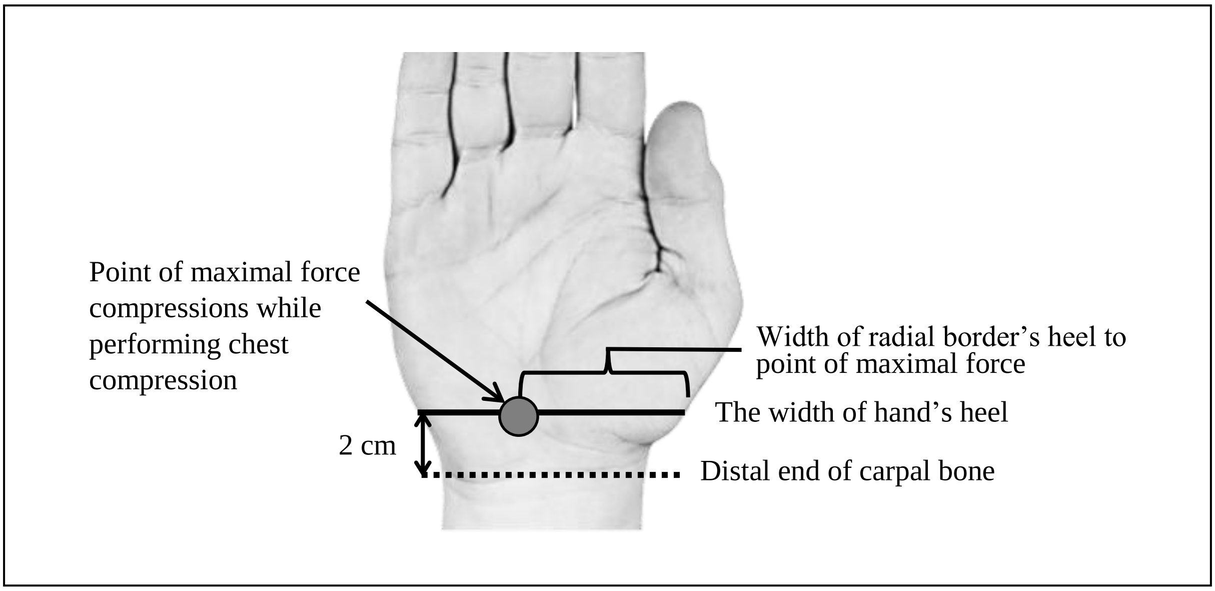

Figure S1 Rescuer’s Hand Measurement

Abbreviations: *cm*, centimeter.

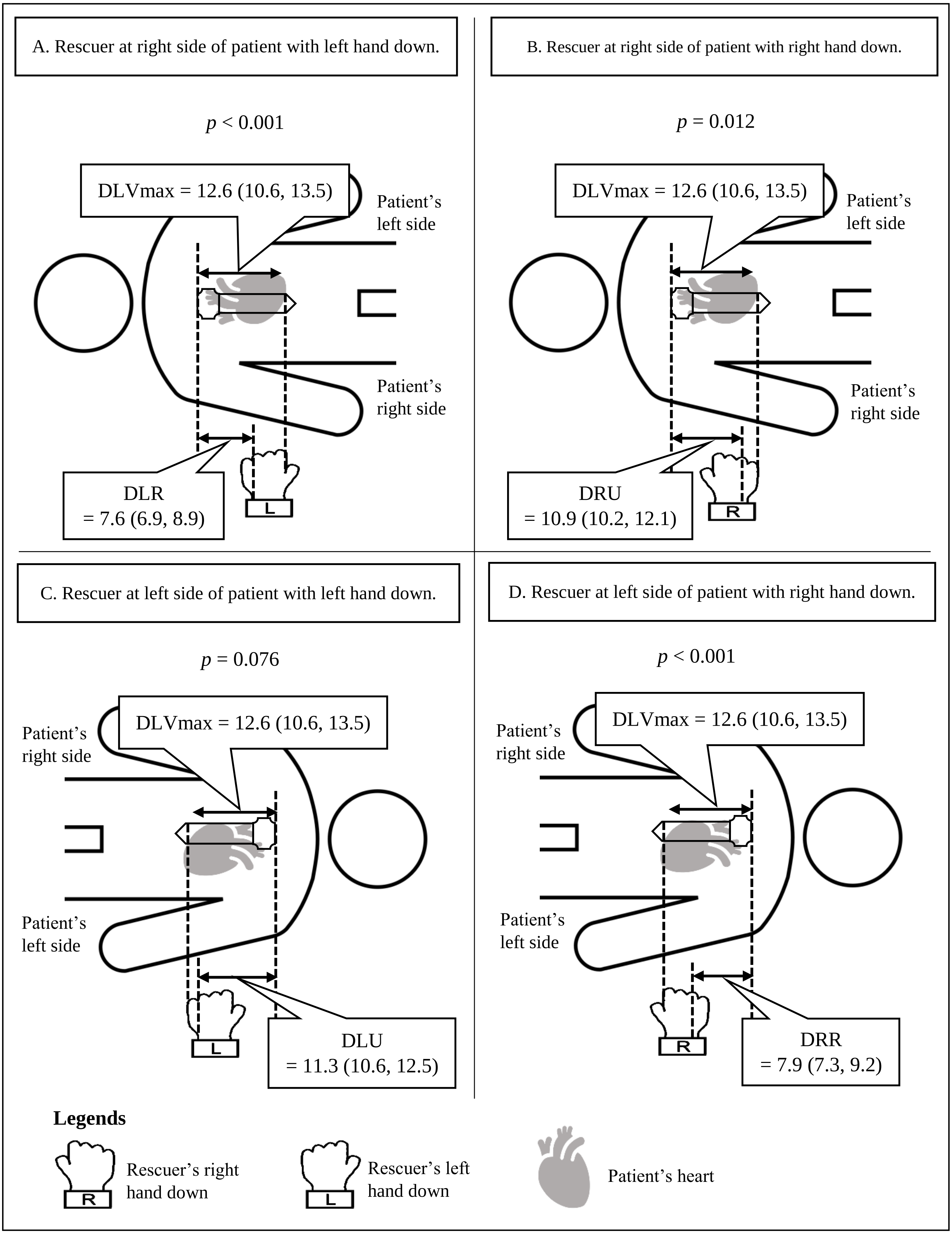
A. Rescuer at right side of patient with left hand down.
p < 0.001
DLVmax = 12.6 (10.6, 13.5)
Patient's left side
Patient's right side
DLR = 7.6 (6.9, 8.9)
L
B. Rescuer at right side of patient with right hand down.
p = 0.012
DLVmax = 12.6 (10.6, 13.5)
Patient's left side
Patient's right side
DRU = 10.9 (10.2, 12.1)
R
C. Rescuer at left side of patient with left hand down.
p = 0.076
DLVmax = 12.6 (10.6, 13.5)
Patient's right side
Patient's left side
DLU = 11.3 (10.6, 12.5)
L
D. Rescuer at left side of patient with right hand down.
p < 0.001
DLVmax = 12.6 (10.6, 13.5)
Patient's right side
Patient's left side
DRR = 7.9 (7.3, 9.2)
R
Legends
R
Rescuer's right hand down
L
Rescuer's left hand down
Patient's heart

Figure S2 Optimal Landmarks for Chest Compressions. The distances showed as median (interquartile range) in centimeters. Differences were analyzed with Wilcoxon signed-rank test.

Abbreviations: *DLVmax*, distance from the sternal notch to the point of maximal left ventricular diameter on the sternum; *DLR*, distance from the sternal notch to the point of maximal force of the rescuer's left hand with the radial border located at the xiphisternal junction; *DLU*, distance from the sternal notch to the point of maximal force of the rescuer's left hand with the ulnar border located at the xiphisternal junction; *DRR*, distance from the sternal notch to the point of maximal force of the rescuer's right hand with the radial border located at the xiphisternal junction; *DRU*, distance from the sternal notch to the point of maximal force of the rescuer's right hand with the ulnar border located at the xiphisternal junction; *95% CI*, 95% confidence interval.

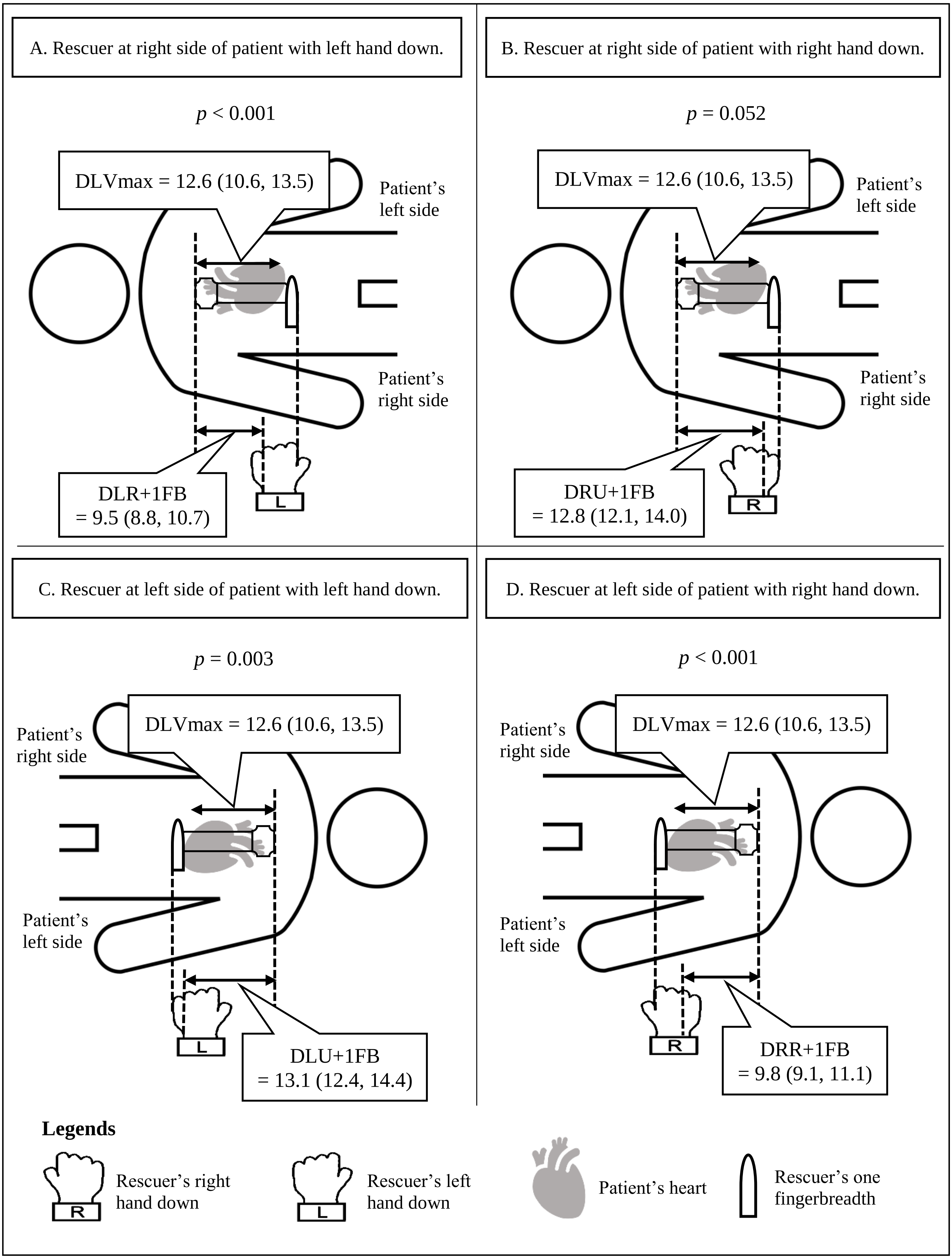

A. Rescuer at right side of patient with left hand down.
p < 0.001
DLVmax = 12.6 (10.6, 13.5)
Patient's left side
Patient's right side
DLR+1FB = 9.5 (8.8, 10.7)
L
B. Rescuer at right side of patient with right hand down.
p = 0.052
DLVmax = 12.6 (10.6, 13.5)
Patient's left side
Patient's right side
DRU+1FB = 12.8 (12.1, 14.0)
R
C. Rescuer at left side of patient with left hand down.
p = 0.003
DLVmax = 12.6 (10.6, 13.5)
Patient's right side
Patient's left side
L
DLU+1FB = 13.1 (12.4, 14.4)
D. Rescuer at left side of patient with right hand down.
p < 0.001
DLVmax = 12.6 (10.6, 13.5)
Patient's right side
Patient's left side
R
DRR+1FB = 9.8 (9.1, 11.1)
Legends
R
Rescuer's right hand down
L
Rescuer's left hand down
Patient's heart
Rescuer's one fingerbreadth

Figure S3 Optimal Landmark for Chest Compressions: Add one fingerbreadth below the xiphisternal junction. The distances showed as median (interquartile range) in centimeter. Differences were analyzed with Wilcoxon signed-rank test.

Abbreviations: *DLVmax*, distance from the sternal notch to the point of maximal left ventricular diameter on the sternum; *DLR + 1FB*, distance from the sternal notch to the point of maximal force of the rescuer's left hand with the radial border located at below the xiphisternal junction one fingerbreadth; *DLU + 1FB*, distance from the sternal notch to the point of maximal force of the rescuer's left hand with the ulnar border located at below the xiphisternal junction one fingerbreadth; *DRR + 1FB*, distance from the sternal notch to the point of maximal force of the rescuer's right hand with the radial border located at below the xiphisternal junction one fingerbreadth; *DRU + 1FB*, distance from the sternal notch to the point of maximal force of the rescuer's right hand with the ulnar border located at below the xiphisternal junction one fingerbreadth; *95% CI*, 95% confidence interval.

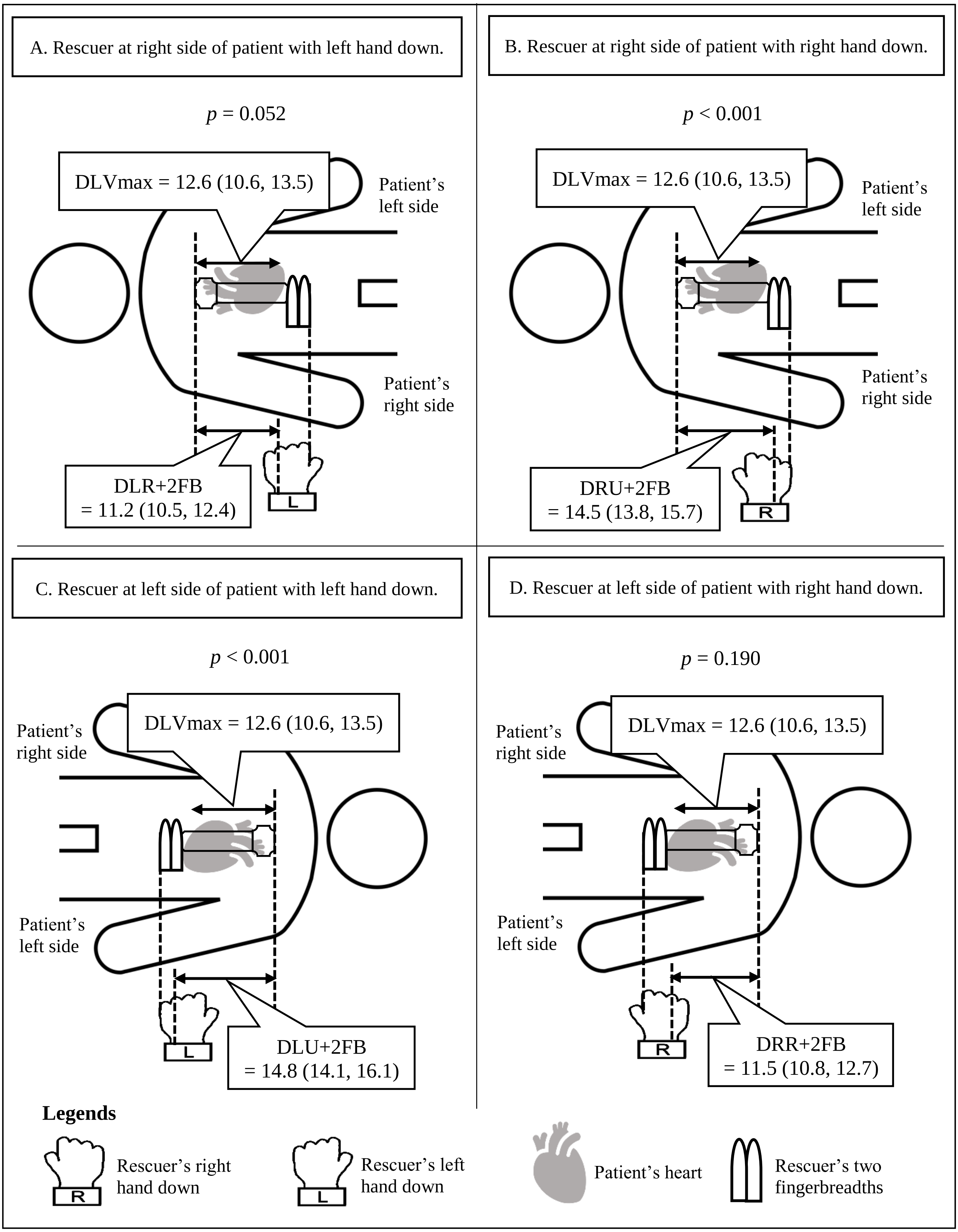

A. Rescuer at right side of patient with left hand down.
p = 0.052
DLVmax = 12.6 (10.6, 13.5)
Patient's left side
Patient's right side
DLR+2FB = 11.2 (10.5, 12.4)
L
B. Rescuer at right side of patient with right hand down.
p < 0.001
DLVmax = 12.6 (10.6, 13.5)
Patient's left side
Patient's right side
DRU+2FB = 14.5 (13.8, 15.7)
R
C. Rescuer at left side of patient with left hand down.
p < 0.001
DLVmax = 12.6 (10.6, 13.5)
Patient's right side
Patient's left side
DLU+2FB = 14.8 (14.1, 16.1)
L
D. Rescuer at left side of patient with right hand down.
p = 0.190
DLVmax = 12.6 (10.6, 13.5)
Patient's right side
Patient's left side
DRR+2FB = 11.5 (10.8, 12.7)
R
Legends
Rescuer's right hand down
Rescuer's left hand down
Patient's heart
Rescuer's two fingerbreadths

Figure S4 Optimal Landmark for Chest Compressions: Add two fingerbreadths below the xiphisternal junction. The distances showed as median (interquartile range) in centimeter. Differences were analyzed with Wilcoxon signed-rank test.

Abbreviations: *DLVmax*, distance from the sternal notch to the point of maximal left ventricular diameter on the sternum; *DLR + 2FB*, distance from the sternal notch to the point of maximal force of the rescuer's left hand with the radial border located at below the xiphisternal junction two fingerbreadths; *DLU + 2FB*, distance from the sternal notch to the point of maximal force of the rescuer's left hand with the ulnar border located at below the xiphisternal junction two fingerbreadths; *DRR + 2FB*, distance from the sternal notch to the point of maximal force of the rescuer's right hand with the radial border located at below the xiphisternal junction two fingerbreadths; *DRU + 2FB*, distance from the sternal notch to the point of maximal force of the rescuer's right hand with the ulnar border located at below the xiphisternal junction two fingerbreadths; *95% CI*, 95% confidence interval.

A. Rescuer at left side of patient with left hand down. Locate the ulnar border of left hand at the xiphisternal junction.

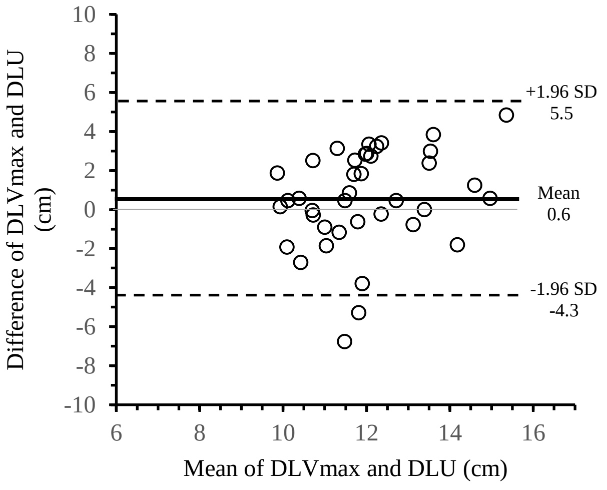

B. Rescuer at right side of patient with right hand down. Locate the ulnar border of right hand at below the xiphisternal junction one fingerbreadth.

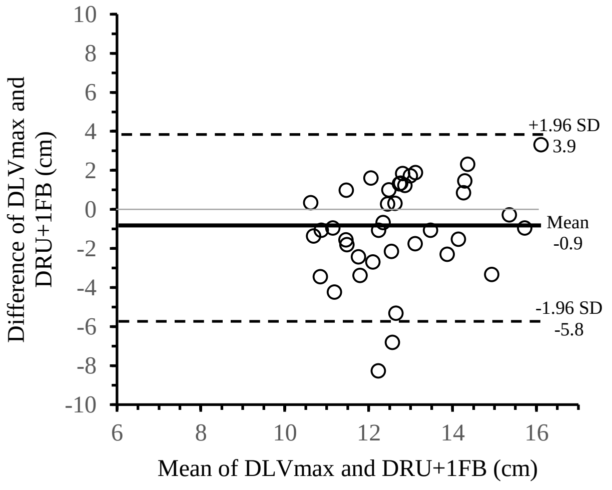

C. Rescuer at right side of patient with left hand down. Locate the radial border of left hand at below the xiphisternal junction two fingerbreadths.

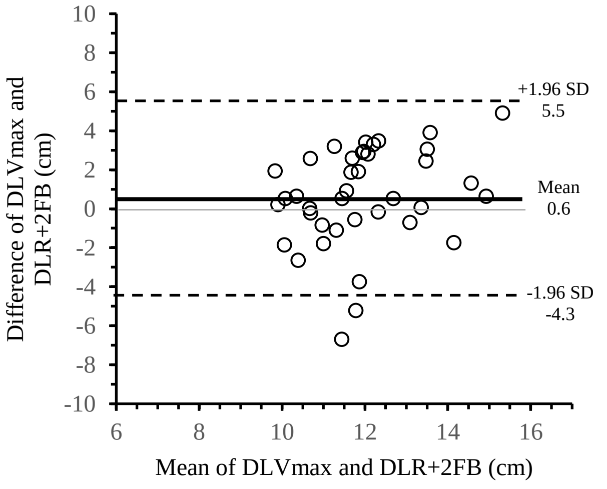

D. Rescuer at left side of patient with right hand down. Locate the radial border of right hand at below the xiphisternal junction two fingerbreadths.

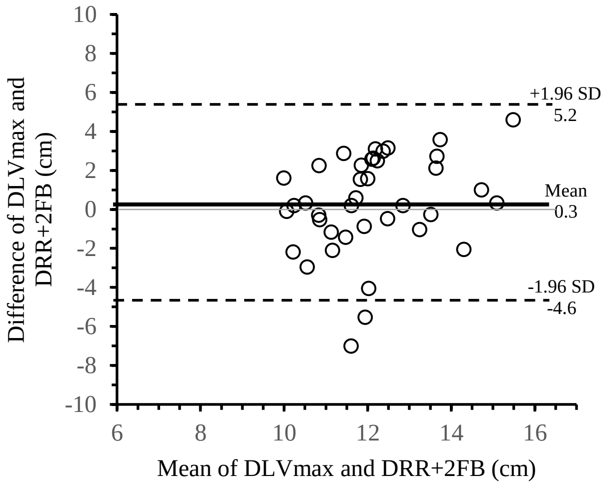

Figure S5 Summary of the Optimal Landmark for Chest Compressions by Modified Bland-Altman Plots. Horizontal axes are mean of DLVmax and DLU, DRU + 1FB, DLR + 2FB or DRR + 2FB. Vertical axes are differences of DLVmax and DLU, DRU + 1FB, DLR + 2FB or DRR + 2FB. Horizontal solid lines are mean of those differences. Two dashed lines are

+1.96 and -1.96 of standard deviations of those differences. Shapiro-Wilk test is checked to ensure normal distribution of those differences and able to use standard deviation to define limits of agreement.

Abbreviations: *DLVmax*, distance from the sternal notch to the point of maximal left ventricular diameter on the sternum; *DLU*, distance from the sternal notch to the point of maximal force of the rescuer's left hand with the ulnar border located at the xiphisternal junction; *DRU + 1FB,* distance from the sternal notch to the point of maximal force of the rescuer's right hand with the ulnar border located at below the xiphisternal junction one fingerbreadth; *DLR + 2FB,* distance from the sternal notch to the point of maximal force of the rescuer's left hand with the radial border located at below the xiphisternal junction two fingerbreadths; *DRR + 2FB*, distance from the sternal notch to the point of maximal force of the rescuer's right hand with the radial border located at below the xiphisternal junction two fingerbreadths; *cm*, centimeter; *SD*, standard deviation.
